# Advanced HCC with amplified mesenchymal epithelial transition factor receptor responds well to savolitinib: a case report

**DOI:** 10.3389/fmed.2023.1130012

**Published:** 2023-05-24

**Authors:** Ningning Yan, Ziheng Zhang, Sanxing Guo, Shujing Shen, Xingya Li

**Affiliations:** ^1^Department of Medical Oncology, The First Affiliated Hospital of Zhengzhou University, Zhengzhou, China; ^2^Department of Radiation Oncology, The First Affiliated Hospital of Zhengzhou University, Zhengzhou, China

**Keywords:** liver cancer, tyrosine kinase inhibitors, MET, savolitinib, MET-TKI

## Abstract

**Objective:**

Current treatment agents for HCC are mostly immune checkpoint inhibitors (ICIs) plus bevacizumab and multitarget tyrosine kinase inhibitors (TKIs); however, their limited overall response rate and shorter median progression-free survival (PFS) discourage their frequent use. The development of Mesenchymal Epithelial Transition Factor receptor (MET) Tyrosine Kinase Inhibitors (MET-TKI) has transformed the treatment pattern in MET-altered solid tumors and improved their prognosis. However, the benefits of MET-TKIs in MET-amplified hepatocellular carcinoma (HCC) remain unclear.

**Methods:**

Here, we present a case of advanced HCC amplified with MET treated with savolitinib, a MET-TKI, after progression from first-line treatment with bevacizumab plus sintilimab.

**Results:**

The patient achieved a partial response (PR) to savolitinib in the second line setting. The progression-free survival (PFS) of first-line of bevacizumab plus sintilimab and sequential second-line treatment with MET-TKI, savolitinib, are 3 and over 8 months, respectively. furthermore, the patient still had continuous PR status with manageable toxicities.

**Conclusions:**

The present case report provides first-hand evidence that savolitinib may be beneficial for patients with advanced MET-amplified HCC and offers a promising treatment option.

## Introduction

In 2020, Hepatocellular carcinoma (HCC) was the sixth most diagnosed cancer and the third leading cause of cancer-related mortality worldwide (1). HCC is the most common type of primary liver cancer and comprises 75 to 85% of all cases (1). The 5-year survival rate of patients with advanced HCC is <5%. Recently, the benefits of novel agents have been attributed to decreased HCC-related mortality.

Immune checkpoint inhibitors (ICI) plus bevacizumab are the standard treatment for HCC in the first-line setting (2, 3). Prolonged overall survival (OS) is ~2 years. However, the objective response rate was <40%. Second-line treatment options are limited and efficacy is poor. Therefore, the development of novel strategies is urgently needed. With the development of sequencing technology, more driver oncogenes have been identified in solid tumors. The mesenchymal epidermal transforming factor receptor (MET) gene encodes a receptor tyrosine kinase (RTK) for hepatocyte growth factor (HGF), which activates downstream signaling pathways involved in tumor progression and metastasis (4). The genomic status of MET, including mutations, amplifications, and fusions, can lead to clinically relevant oncogenesis. MET amplification approximately accounts for 2–6% of advanced HCC patients (5). Therefore, c-MET has been considered an oncogenic driver for HCC, and a previous report has found that c-MET activity is involved in resistance to sorafenib in HCC (6).

A previous phase 2 study demonstrated that MET-TKI tivantinib could improve the prognosis of advanced HCC in a second-line setting (7). However, the confirmed phase 3 trial could not replicate the results of tivantinib in a phase 2 study (8). Savolitinib is a highly selective MET inhibitor and shows great efficacy in MET exon 14 skipping mutations and MET amplification in treatment of non-small cell lung cancer (NSCLC) (9, 10). However, to our knowledge, no reports have documented the potential of savolitinib in MET-amplified HCC. Here, we present a case of advanced HCC with amplification of the c-MET gene administered with savolitinib as a second-line treatment. After 2 months of savolitinib treatment, the patient achieved a partial response and the serum tumor marker level dropped drastically.

## Case presentation

**Figure 1:**
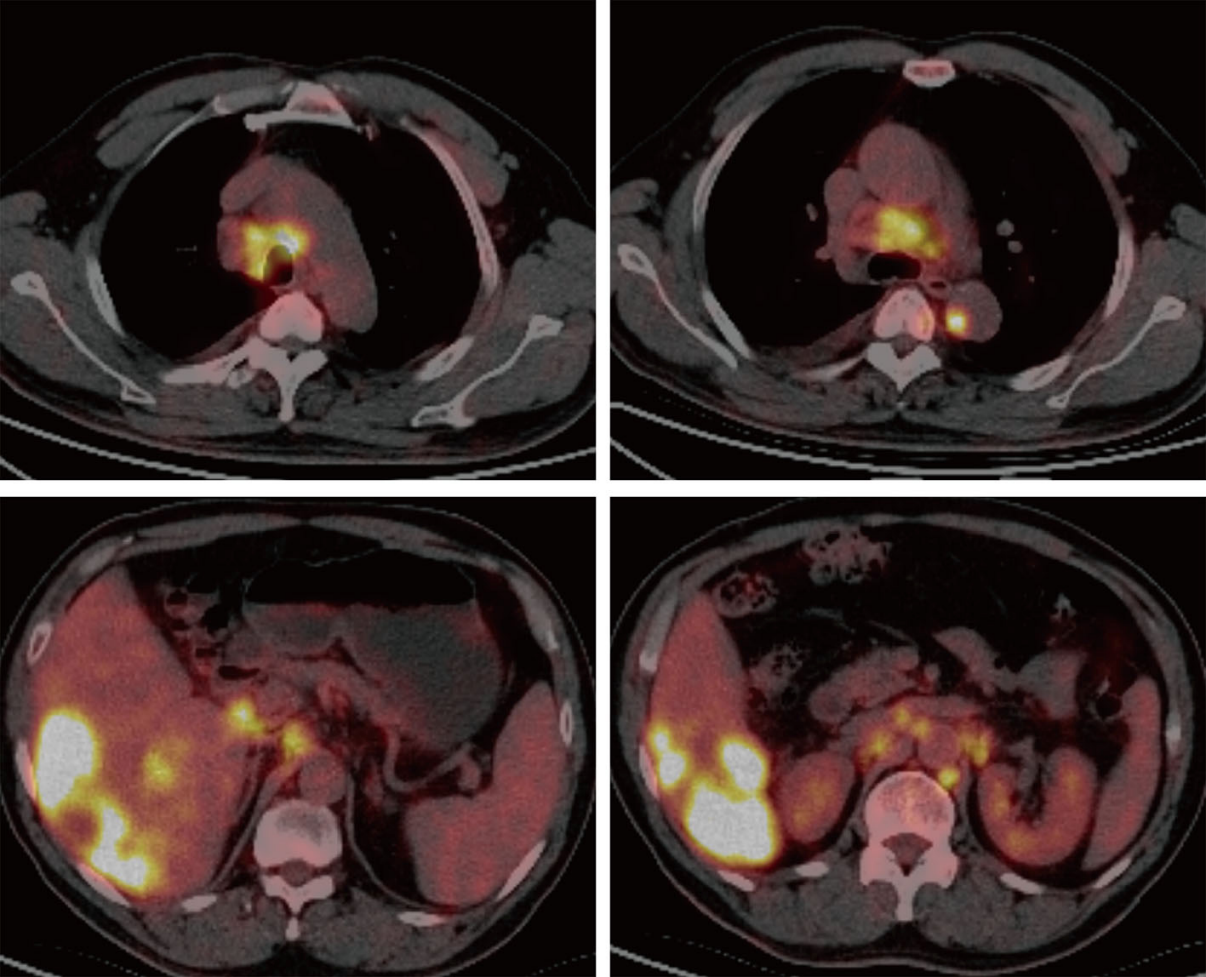
PET/CT imaging. PET/CT revealed a low-density shadow in the right liver lobe with multiple nodes in the liver, bilateral supraclavicular and subclavian lymph nodes, mediastinal lymph nodes, lymph nodes on the left side of the spine, celiac lymph nodes, retroperitoneal lymph nodes, and vertebral body metastases.

**Figure 2:**
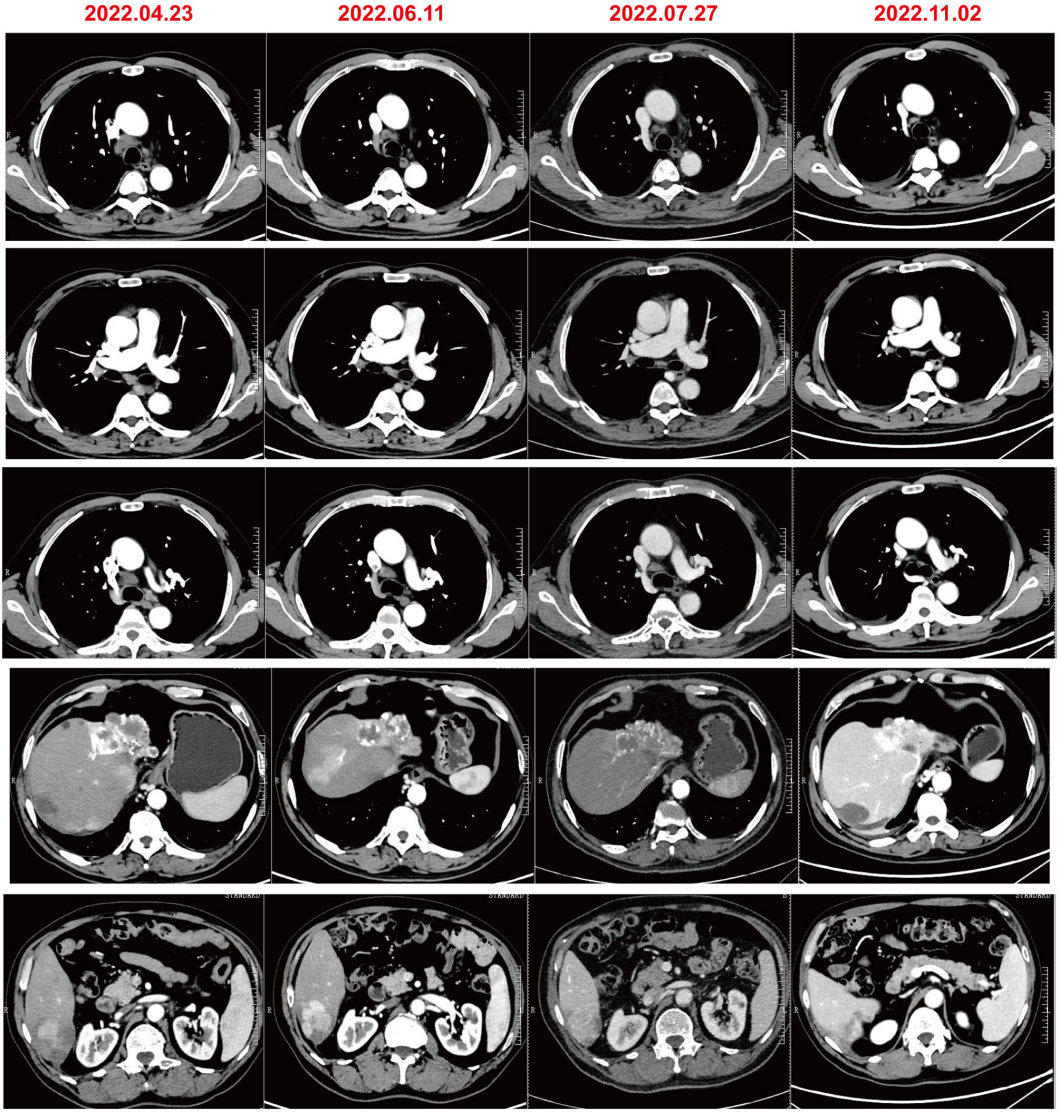
Evaluation of radiographic response evaluation.

**Figure 3:**
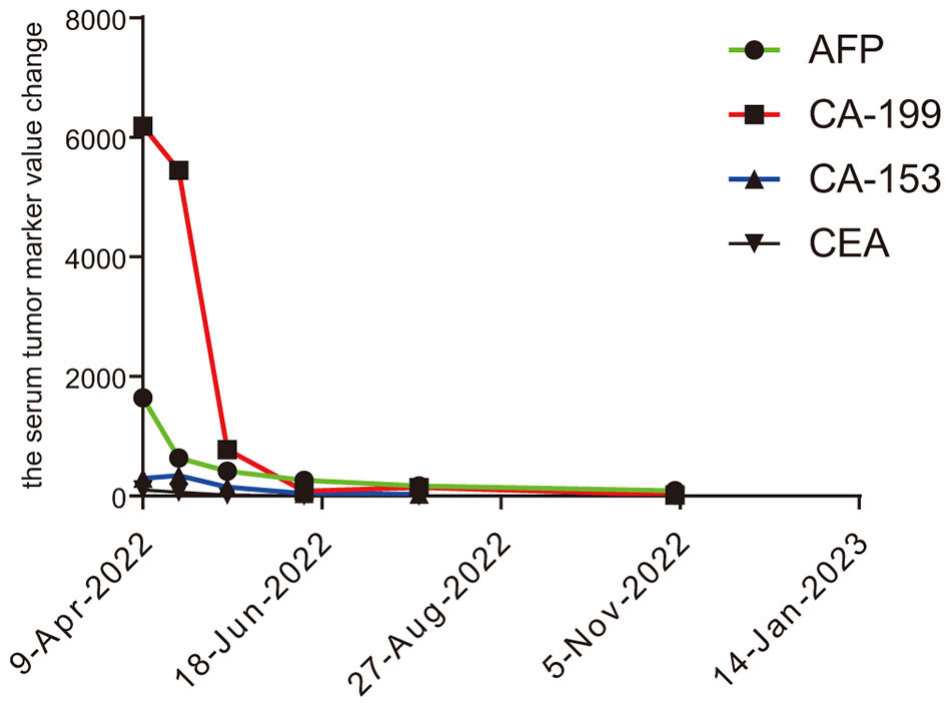
Changes recorded in tumor serum markers, including AFP, CA19-9, CA15-3, and CEA.

A 61-year-old male patient with severe pain in the left abdomen was admitted to our hospital, the First affiliated Hospital of Zhengzhou University, on January 9, 2022. The patient had a 12-year history of hepatitis and was taking antiviral agents regularly. PET/CT revealed a low-density shadow in the right liver lobe with multiple nodes in the liver, bilateral supraclavicular and subclavian lymph nodes, mediastinal lymph nodes, lymph nodes on the left side of the spine, celiac lymph nodes, retroperitoneal lymph nodes, and vertebral body metastases (Figure 1). A liver biopsy revealed poorly differentiated HCC. Hence, the patient was diagnosed with stage IV HCC with Child-Pugh class A at the initial diagnosis. The patient then received sintilimab (200 mg, d1) plus bevacizumab (15 mg/m^2^) for one cycle. However, as serum tumor marker levels increased, a CT was performed, which revealed stable disease according to the RECIST 1.1 guidelines; accordingly, two cycles of sintilimab (200 mg, d1) plus bevacizumab (15 mg/m^2^) were continued. Due to the continuous elevation of serum tumor markers including AFP, CA19-9, CA15-3 and CEA, disease progression was confirmed with only 3 months progression free survival (PFS). Next-generation sequencing performed on the specimen at initial diagnosis revealed MET amplification (24.7 folds higher than upper normal limits). In addition, some missense mutations including oncogene FGFR2, JAK3, RHOA and TP53 frameshift mutation were meaningless, no approved drugs targeting them were available for now (Table 1). Previous studies have shown that patients with high MET amplification level (gene copy number ≥5 or MET to CEP7 ratio ≥2 by FISH or NGS) were more likely to benefit from MET inhibitors (11, 12).Therefore, we switched to Savolitinib, which is a highly selective MET-TKI. After 7 days, serum tumor marker levels decreased drastically and a CT scan confirmed partial response (PR) (Figures 2, 3; Table 2). The patient continued to show PR for over 8 months. In addition, the patient reported grade 2 gastrointestinal effects according to CTCAE grading, including belching and abdominal pain, the symptoms were relieved when administered with proton pump inhibitors. Grade 1 Edema occurred in both legs and only physical therapy including elevating legs, diet control, and wearing stretch socks were administered. No dose reduction or interruption was done to control these adverse events.

**Table 1:** Summary of genomic alterations tested using NGS.

**Gene names**	**Transcript code**	**EXON**	**Results**	**Abundance**	**Alteration type**
MET	NM_000245.4		MET copy number amplification	CN:24.7	Gene amplification
TP53	NM_000546.5	4	p. P72Lfs*76	42.0%	Frameshift mutation
FGFR2	NM_000141.4	13	p. S605P	22.5%	Missense mutation
JAK3	NM_000215.3	13	p. V585L	45.8%	Missense mutation
RHOA	NM001664.4	2	p. V9M	22.6%	Missense mutation

**Table 2:** The change of serum tumor markers.

**Tumor maker**	**2022.04.09**	**2022.06.18**	**2022.08.27**	**2022.11.05**	**2023.01.14**
AFP	1,642	635	414	264	89
CA19-9	6,190	5,450	774	81.9	16.9
CA15-3	296	340	150	34.3	13.7
CEA	104	60.3	18	3.98	2.67

## Discussion

More than 50% of newly diagnosed HCC cases and mortalities are from China cause of the largest population living with Hepatitis B virus^1^. According to statistics, ~50–60% of cases of HCC ultimately require treatment with systemic therapies (1). The first-line setting of HCC has improved significantly with molecular therapies such as sorafenib, lenvatinib and donafenib, while the second-line setting has only achieved 8–11 months of overall survival (13, 14). However, with the advent of immunotherapies, ICI plus anti-vascular endothelial growth factor antibodies have shown superior efficacy compared to sorafenib in patients with advanced stage HCC, leading to a new first-line median OS duration of ~2 years (2, 3). If the first line of treatment fails, there are limited options available. Therefore, the development of novel treatment strategies is urgently required.

Here, we present a case of advanced HCC that harbors MET amplification. Serum tumor markers, AFP, CA19-9, and CA15-3, were drastically elevated after three cycles of sintilimab plus bevacizumab, and radiological evaluation confirmed inefficacy. Therefore, we chose savolitinib, a selective MET inhibitor, as second-line treatment and achieved a better response, with serum tumor makers including AFP, CA19-9, and CA15-3 showing a marked decrease. In addition, a PFS of over 8 months was observed and the case was still in PR status.

Savolitinib has been developed to treat metastatic NSCLC, clear and papillary renal cell carcinoma (RCC), gastric cancer, and colorectal cancer with MET alterations (15). A previous study revealed that savolitinib has antitumor efficacy in MET-altered gastric cancer patient-derived tumor xenograft (PDX) models (16). Additionally, a recent report demonstrated that the response was observed only observed in gastric cancers (17). However, in NSCLCs, patients with c-MET 14 exon skipping and MET amplification responded to savolitinib (10). Our results, combined with these data, may provide clues why our case showed a response to savolitinib. In another study, a previous report suggested that patients with MET amplification could narrowly benefit from immunotherapy (18); this might indicate that the case presented a poor response to ICI plus anti-VEGF inhibitors.

## Conclusion

In summary, it is reasonable to choose savolitinib to treat HCC patients with positive c-MET amplification, and the profile of adverse events is manageable. Furthermore, the c-MET gene test should be performed routinely in patients with HCC. However, since this was only a case report, more randomized clinical trials, including larger sample sizes, were conducted to obtain concrete insights. Our case shows that savolitinib is suitable for treating HCC in patients with MET amplification.

## Data availability statement

The original contributions presented in the study are included in the article/supplementary material, further inquiries can be directed to the corresponding authors.

## Ethics statement

Written informed consent was obtained from the participant/patients for the publication of this case report.

## Author contributions

NNY, ZHZ, SJS, and XYL wrote the manuscript. NNY, ZHZ, and SXG prepared Figures and Tables. All authors have reviewed the manuscript and gave their consent to publication. All authors contributed to the article and approved the submitted version.
