# Corrigendum: Advanced HCC with amplified mesenchymal epithelial transition factor receptor responds well to savolitinib: a case report

**DOI:** 10.3389/fmed.2023.1256685

**Published:** 2023-07-26

**Authors:** Ningning Yan, Ziheng Zhang, Sanxing Guo, Shujing Shen, Xingya Li

**Affiliations:** ^1^Department of Medical Oncology, The First Affiliated Hospital of Zhengzhou University, Zhengzhou, China; ^2^Department of Radiation Oncology, The First Affiliated Hospital of Zhengzhou University, Zhengzhou, China

**Keywords:** liver cancer, tyrosine kinase inhibitors, MET, savolitinib, MET-TKI

In the published article, the author names were incorrectly written as “Ning Yan, Zi Zhang, San Guo, Shu Shen, Xing Li”. The correct spellings are “Ningning Yan, Ziheng Zhang, Sanxing Guo, Shujing Shen, Xingya Li”.

In the published article, there was a mistake in the Author contributions. The correct Author contributions appears below.

## Author contributions

NNY, ZHZ, SJS, and XYL wrote the manuscript. NNY, ZHZ, and SXG prepared Figures and Tables. All authors have reviewed the manuscript and gave their consent to publication. All authors contributed to the article and approved the submitted version.

The authors apologize for these errors and state that this does not change the scientific conclusions of the article in any way. The original article has been updated.

